# Serial Surveillance of Viable Bacterial Contamination in a German Hotel Across Pre-COVID and Pandemic Recovery Periods: Persistent Hotspots and Functional-Area Heterogeneity

**DOI:** 10.3390/microorganisms14102183

**Published:** 2026-09-29

**Authors:** Alexander Martens, Markus Schauer, Mohamad Motevalli, Susanne Mair, Brigitte König

**Affiliations:** 1 Institute for Medical Microbiology and Virology, University of Leipzig Medical Center, 04103 Leipzig, Germany; 2 Limbach Research Institute for Preventive Diagnostics and Healthy Aging, German University of Health & Sport (DHGS), 85737 Ismaning, Germany; markus.schauer@dhgs-hochschule.de (M.S.); moha.motevalli@dhgs-hochschule.de (M.M.); susanne.mair.dz@edu.dhgs-hochschule.de (S.M.)

**Keywords:** microorganisms, built environment, environmental microbiology, hospitality hygiene, surface contamination, culturable bacteria, high-touch surfaces, persistent contamination, infection prevention, COVID-19

## Abstract

Hotels contain diverse functional environments, yet longitudinal evidence on viable bacterial contamination and its response to COVID-associated hygiene changes remains limited. This retrospective serial cross-sectional study analyzed 544 environmental samples from five audits of a German hotel conducted in 2016, 2018, 2019, 2022, and 2023. Pre-pandemic audits (2016, 2018–2019) were compared with 2022–2023 audits across four functional environments: guest rooms, child-oriented areas, wellness/water facilities, and food-service operations. Outcomes included culture positivity, contamination severity, polymicrobial and priority-microorganism detection, persistent hotspots, and organism distributions. Modified Poisson models adjusted for functional environment and used cluster-robust inference. All statistical analyses were performed in R (version 4.5.1). High contamination declined from 54.2% pre-COVID to 19.3% in 2022–2023 (aPR, 0.36; 95% CI, 0.25–0.54; *p* < 0.001), an adjusted reduction of 34.1 percentage points (95% CI, 21.5–46.6). Culture positivity remained unchanged (63.4% vs. 60.1%; aPR, 0.96; 95% CI, 0.83–1.12; *p* = 0.634), whereas polymicrobial contamination decreased (aPR, 0.61; 95% CI, 0.39–0.96; *p* = 0.032; adjusted reduction, 8.3 percentage points; 95% CI, 1.0–15.7). Priority-microorganism detection was unchanged (aPR, 1.06; 95% CI, 0.67–1.67; *p* = 0.804), despite a shift toward *S. aureus* and *P. aeruginosa*. Culture positivity remained approximately 90% in child-oriented and food-service areas. Eight of 15 recurring surface categories remained persistent hotspots, including wet-room floors, frequently touched controls, paddling-pool features, and food-service machinery. The *Bacillus cereus* group decreased from 12.6% to 2.5%, whereas *Staphylococcus aureus* and *Pseudomonas aeruginosa* were each detected in 0% versus 5.8% of samples (*q* < 0.001). Audits in 2022–2023 showed lower contamination intensity without corresponding reductions in overall culture positivity or priority-microorganism prevalence. Complementary burden-, complexity-, and organism-based indicators are needed for environmental-hygiene evaluation.

## 1. Introduction

The hospitality sector constitutes a distinctive component of the built environment, bringing together large and continually changing populations within spaces characterized by intensive surface contact, shared amenities, and diverse operational activities [1,2,3]. In hotels, microorganisms introduced by guests, employees, food handling, water, outdoor air, footwear, and cleaning equipment can be deposited on guest-room furnishings, sanitary fixtures, door and device controls, recreational installations, and food-service machinery. Depending on the material, moisture, nutrient availability, contact frequency, and interval between cleaning cycles, these surfaces may support transient contamination or function as recurrent environmental reservoirs [3,4,5,6]. Environmental microbiological surveillance is therefore an important element of hygiene assurance in accommodation facilities, complementing visual inspection by identifying where viable contamination persists and where cleaning and disinfection resources may require more focused deployment.

Despite the scale of the hospitality industry, environmental contamination in hotels has received far less attention than contamination in healthcare facilities and other institutional settings. Available evidence indicates that frequently touched surfaces in accommodation environments can harbor heterogeneous bacterial populations, including common human- and environment-associated organisms, hygiene indicators, and opportunistic microorganisms [3,6,7,8]. Their distribution reflects a dynamic interaction among human traffic, occupant turnover, hand contact, moisture exposure, organic residues, equipment design, surface properties, and the effectiveness of routine hygiene practices [4,5,6,8]. Consequently, contamination is unlikely to be uniform across an entire property. Studies restricted to a small set of touchpoints or a single sampling occasion offer only a partial representation of this heterogeneity and provide limited insight into whether repeatedly contaminated surfaces remain stable over time [3,7,9].

This spatial heterogeneity is particularly relevant in family-oriented hotels, where operational areas with fundamentally different contamination pressures coexist within the same facility [10,11]. Guest rooms and sanitary areas experience rapid turnover and repeated contact with handles, switches, remote controls, and wet room surfaces [3,11,12]. Child-oriented spaces combine dense interaction, frequent hand-to-surface and hand-to-mouth behavior, and extensive use of shared toys, play equipment, and controls [13,14]. Wellness and water-associated areas provide moist ecological niches in which microbial survival, surface recolonization, and biofilm development may be favored, particularly on complex or continuously wet installations [15,16]. Food-service environments introduce additional pressures related to food residues, repeated manipulation, temperature variation, and machinery whose joints, dispensing components, or internal contact surfaces may be difficult to clean uniformly [17,18,19]. These contrasts make functional-area analysis essential: property-wide averages may conceal operational zones or surface types that contribute disproportionately to the observed microbial burden.

Robust environmental-hygiene assessment also requires more than a binary distinction between positive and negative cultures. Culture positivity describes the frequency with which viable microorganisms are recoverable, whereas colony burden reflects the intensity of contamination and may respond differently to hygiene measures [20,21]. The surface can remain culture-positive after cleaning while carrying a substantially reduced microbial load; conversely, a relatively small number of recurrent sites may retain high contamination and continue to dominate the hygiene profile of a facility [7,21]. Polymicrobial recovery provides further information about the complexity of contamination, while the detection of priority microorganisms and fecal indicators adds organism-specific hygienic relevance [20,22,23]. When these measures are combined with repeated, surface-resolved surveillance, they allow differentiation between widespread low-level microbial persistence, episodic high-burden contamination, stable surface hotspots, and shifts in the organisms composing the recoverable microbiota.

The COVID-19 pandemic produced an exceptional change in environmental-hygiene practice across the hospitality sector [24,25]. Cleaning and disinfection frequencies were increased, high-touch surfaces received greater attention, hand-hygiene measures were expanded, and housekeeping and shared-space procedures were reorganized [24,25]. Although these interventions were implemented primarily to reduce viral transmission, their broader influence on culturable bacterial contamination is of considerable environmental-microbiological interest [21,26,27]. Intensified hygiene could reduce colony burden without eliminating continual microbial deposition, and the magnitude of change could vary among dry touch surfaces, wet installations, child-use equipment, and food-related machinery [7,26]. At the same time, altered occupancy, guest behavior, and operational routines may have changed the pattern of microbial introduction and redistribution within hotels [4,25,28]. However, environmental research conducted during the pandemic focused primarily on SARS-CoV-2 detection or short-term assessments, leaving limited evidence on how viable bacterial contamination, polymicrobial recovery, persistent surface hotspots, and organism profiles differed from established pre-pandemic conditions [29,30].

The availability of serial audits from the same hotel in the pre-pandemic years (2016, 2018, and 2019) and in 2022–2023 allowed temporal differences in environmental contamination to be examined within a consistent hospitality setting. The present study evaluated five environmental-hygiene audits undertaken between 2016 and 2023 in a hotel in Bavaria, Germany, encompassing guest rooms, child-oriented areas, wellness and water-associated facilities, and food-service operations. The study aimed to determine whether the 2022–2023 period differed from the pre-COVID period in the prevalence and severity of culturable surface contamination and in the occurrence of polymicrobial and priority-microorganism contamination. It further examined whether temporal patterns varied across functional environments, which recurring surface categories constituted persistent contamination hotspots, and whether the distribution of selected microorganisms and broader taxonomic groups changed between surveillance periods. The findings are intended to strengthen risk-informed environmental-hygiene surveillance and guide targeted cleaning, disinfection, and microbiological monitoring in hospitality settings.

## 2. Materials and Methods

### 2.1. Study Design and Sampling Framework

This retrospective observational study analyzed serial cross-sectional environmental-hygiene audits conducted at a hotel in Bavaria, Germany. The source material comprised five audit campaigns performed in 2016, 2018, 2019, 2022, and 2023. Each campaign was treated as a separate cross-sectional assessment. The hotel was selected based on available serial environmental-hygiene audit records. Its family-oriented facilities provided access to diverse functional environments within one property. The principal comparison contrasted pre-pandemic audits (2016, 2018, and 2019) with audits conducted in 2022–2023. No audit data were available for 2020–2021; therefore, this comparison does not assess changes during the initial pandemic response. During this interval, the hotel experienced closures and restricted guest intake, with wellness facilities largely closed. A complementary three-level phase variable distinguished the initial audit (2016), pre-COVID follow-up (2018–2019), and follow-up in 2022–2023.

The original audits targeted hygiene-relevant and frequently contacted surfaces across guest accommodation, child-oriented facilities, wellness and water-associated areas, and food-related operations. Examples included sanitary fixtures, floors and wet-room surfaces, door handles, light switches, remote controls, toys and shared-play equipment, pool or slide components, and food-service equipment. Across all audits, 283 samples came from 28 guest rooms (6–20 per room), 112 from child-oriented areas (baby club, 23; crawling club, 26; mini club, 31; adventure club, 18; soft-play area, 8; animal-play area, 6), 95 from wellness/water facilities, and 54 from food-service areas (kitchen, 28; buffet, 26). Each audit followed its predefined operational surveillance scope, resulting in year-specific distributions of sampled surfaces. Sampling was conducted under the routine operational conditions of each functional area. Guest rooms, child-oriented areas, and wellness/water facilities were generally sampled after routine cleaning and, where applicable, before opening or while unoccupied. Food-service areas were sampled during operation in relation to their ongoing cleaning schedules. Sites were selected purposively for their hygienic relevance, rather than through simple random or stratified random sampling. Sampling dates within each year were unavailable in the analytical dataset, preventing verification of seasonal consistency across audits. Documented sampling conditions were retained and evaluated in sensitivity analyses addressing comparability between surveillance periods.

### 2.2. Sample Collection, Transport, and Microbiological Culture

All environmental surfaces were sampled using sterile ESwab collection systems (Copan Italia S.p.A., Brescia, Italy) containing liquid Amies transport medium. The medium comprises sodium chloride, potassium chloride, calcium chloride, magnesium chloride, monopotassium phosphate, disodium phosphate, and sodium thioglycollate in distilled water [31]. Under the routine swab protocol, the swab was moistened with transport medium and passed across the accessible target area using horizontal, vertical, and diagonal strokes with consistent pressure [10,27,32]. It was then returned to the transport tube, conveyed to the microbiology laboratory, and processed within 24 h of collection. The same multidirectional swabbing procedure, contact duration, standardized measured sampling area, and calibrated swabbing pressure were used across all five audits. Sampling locations followed each audit’s surveillance scope; recurring surface categories did not necessarily represent identical physical objects across audits.

For ESwab samples, a 10 µL aliquot of transport medium was inoculated onto Mueller–Hinton agar supplemented with 5% sheep blood, mannitol salt agar, and Endo agar to support broad bacterial recovery and selective detection of staphylococci and Gram-negative organisms. Quantitative enumeration used serial tenfold dilutions of the transport medium, with 10 µL aliquots from each dilution plated in triplicate on each culture medium. The same criteria for performing quantitative enumeration and the same dilution and plating procedures were applied across all five audits. Colony growth was recorded as exact counts when enumeration was possible and otherwise by the semi-quantitative or censored terminology used in the laboratory report.

Media were incubated aerobically at 37 °C and examined after approximately 24 h. Plates with limited or delayed growth were re-examined after extended incubation of up to 48–72 h. Colonies with distinct morphology, including differences in size, form, pigmentation, texture, or hemolysis, were selected and subcultured to obtain pure isolates. When multiple morphotypes were present, representative colonies were retained to capture within-sample taxonomic diversity.

### 2.3. Microorganism Identification

Pure bacterial isolates were identified by matrix-assisted laser desorption/ionization time-of-flight mass spectrometry using the VITEK MS system (bioMérieux, Lyon, France) and its clinical-use knowledge base. Identifications were accepted according to the manufacturer’s criteria. The system identifies cultured isolates by comparing their characteristic protein mass spectra with reference profiles in its knowledge base. Its performance for routine bacterial identification has been evaluated in clinical and multicenter studies [33,34]. The same culture-media panel (Mueller–Hinton agar supplemented with 5% sheep blood, mannitol salt agar, and Endo agar) and MALDI-TOF identification platform (VITEK MS) were used across all five audits from 2016 to 2023. Isolates not resolved by mass spectrometry were eligible for 16S rRNA gene sequencing. Presumptive *Staphylococcus aureus* isolates were additionally assessed using latex agglutination for coagulase detection. Organism names used in the analysis reflect the taxonomic resolution documented in the original laboratory reports. Similar culture and identification procedures have been reported in a related study from the same laboratory [10].

Routine laboratory quality assurance included sterility verification of culture media, aseptic sample handling, and negative-control procedures using unused swabs when applicable [35,36]. Quality-review annotations were retained for traceability, and analyses excluding annotated records were included among the prespecified sensitivity analyses. Antimicrobial susceptibility testing was not performed because the audits focused on environmental contamination and organism identification rather than antimicrobial-resistance surveillance.

### 2.4. Data Extraction, Harmonization, and Analytical Population

Information from the original audit and laboratory reports was transcribed into a structured sample-level dataset using unique sample identifiers and links to the source report. Extracted fields included audit year, source-report table, hotel environment, subarea, sampled surface or object, documented sampling condition, collection method, original growth or colony-forming unit (CFU) description, contamination classification, reported microorganisms, and quality-assurance annotations.

Sampling locations were grouped into four functional environments before comparative analysis: guest rooms, child-oriented areas, wellness/water-associated areas, and food service. The analytical population contained 544 samples: 283 from guest rooms, 112 from child-oriented areas, 95 from wellness/water-associated areas, and 54 from food service. Six samples from a cosmetics area were outside the defined analytical scope and were not included in this study. The 544 samples represent surface-level observations within a single hotel, not independent hotel-level replicates; they support within-hotel comparisons but do not establish statistical representativeness of German hotels. Audit year was retained as a categorical variable. Source-report table identifiers were preserved because samples recorded together could share collection, processing, or reporting features; the combination of audit year and source-report table therefore defined the primary clustering unit for regression inference. Sampling subareas were also retained for descriptive analyses and alternative clustering.

### 2.5. Measures, Derived Outcomes, and Classifications

Contamination severity was harmonized into four CFU-based categories: no detectable growth (score 0), low contamination (1–50 CFU; score 1), moderate contamination (51–500 CFU; score 2), and high contamination (>500 CFU or confluent growth; score 3). These study-specific categories harmonized numerical and semi-quantitative laboratory reports into ordered levels of increasing culturable burden; the cutoffs were analytical thresholds, not validated hygiene or infection-risk limits. A related hotel study used the same general ordinal approach, although with a different five-level classification [10].

Six microbiological outcomes were derived. Culture positivity denoted any reported culturable microbial growth. High contamination corresponded to severity score 3, and moderate/high contamination to scores 2 or 3. Polymicrobial contamination required at least two distinct standardized microorganism taxa in the same sample. Fecal-indicator detection was defined as the presence of *Enterococcus* spp. or *Escherichia coli*. The priority-microorganism composite indicated detection of at least one member of the *Bacillus cereus* group, *Staphylococcus aureus*, *Pseudomonas aeruginosa*, *Enterococcus* spp., or *Escherichia coli*. Reported organism names were standardized while preserving the distinction between genus-level and species-level identifications. Thirteen selected taxon indicators were constructed for exploratory period comparisons; these comprised the five components of the priority composite and additional recurring *Acinetobacter*, *Bacillus*, *Staphylococcus*, and *Micrococcus* taxa. To reduce sensitivity to changes in taxonomic reporting resolution across audit years, five broader descriptive groups were also generated: *Bacillus* sensu lato, *Staphylococcus*, *Acinetobacter*, *Pseudomonas*, and *Enterococcus*.

Surface descriptions were standardized within each functional environment to identify recurring environment-specific categories. A category entered the hotspot analysis when it contained at least eight samples overall and was represented in at least three audit years; nonspecific other-surface records were omitted. A persistent hotspot was defined descriptively as at least 50% culture positivity in both surveillance periods with at least three samples available in each period. Categories with sufficient period-specific sampling but less than 50% positivity in either period were classified as nonpersistent, whereas those with fewer than three observations in either period were labeled as having limited support. A period-by-surface cell was additionally marked as high burden when at least 50% of three or more severity-evaluable samples had high contamination.

### 2.6. Statistical Analysis

All statistical analyses and visualizations were performed in R version 4.5.1 (R Foundation for Statistical Computing, Vienna, Austria). Sample characteristics and microbiological outcomes were summarized using counts and percentages by audit year, surveillance phase, period, and functional environment, with outcome-specific evaluable samples as denominators. Wilson 95% confidence intervals were presented for descriptive prevalence estimates where applicable.

Associations between surveillance period and culture positivity, high contamination, moderate/high contamination, priority-microorganism detection, and polymicrobial contamination were estimated using separate modified Poisson models with a log link. Primary models adjusted for functional environment and used heteroskedasticity-consistent type 1 (HC1) sandwich standard errors clustered across 57 audit-year-by-source-table combinations; no additional small-sample corrections or wild cluster bootstrap were applied. Adjusted prevalence ratios and 95% confidence intervals (CIs) were obtained from model contrasts; standardized prevalences and absolute differences were derived from counterfactual predictions and the delta method. Additional models examined three-phase contrasts, environment-specific period effects, and period-by-environment interactions using cluster-robust Wald tests. Estimates based on fewer than 10 clusters were interpreted cautiously.

Prespecified sensitivity analyses assessed alternative audit exclusions, shared sampling-condition support, setting and recurring-surface restrictions, removal of quality-assurance-flagged records, and alternative clustering. Bounding analyses reassigned the seven unavailable severity values and six unidentified positive cultures under both extreme assumptions; leave-one-audit-year-out models assessed campaign influence. Frequencies of 13 selected microorganism indicators were compared between the pre-pandemic period and 2022–2023 using two-sided Fisher exact tests, with Benjamini–Hochberg correction. Organism-specific significance was defined as *q* < 0.05; outcome-model and phase comparisons used nominal, unadjusted *p* < 0.05 and were interpreted as exploratory.

## 3. Results

### 3.1. Contamination Outcomes Across Surveillance Periods and Environments

Environmental hygiene surveillance comprised five irregularly spaced cross-sectional audits conducted in 2016, 2018, 2019, 2022, and 2023, yielding 102, 71, 95, 160, and 116 eligible samples, respectively. The observations encompassed an initial audit in 2016, pre-pandemic follow-up in 2018–2019, and follow-up in 2022–2023. Among 544 environmental samples, 336 (61.8%) yielded microbial growth, while 195/537 samples with interpretable contamination severity (36.3%) showed high contamination (Table 1). Across the predefined surveillance phases, high contamination decreased from 64.9% during the initial 2016 audit to 47.9% during the 2018–2019 follow-up phase and 19.3% during the 2022–2023 follow-up phase. In the pooled period comparison, high contamination was observed in 142/262 pre-COVID samples (54.2%) versus 53/275 2022–2023 samples (19.3%), whereas culture positivity remained comparatively stable at 63.4% and 60.1%, respectively.

High contamination decreased across all functional environments, including guest rooms (46.2% to 8.1%), child-oriented areas (72.5% to 36.2%), wellness/water areas (42.9% to 17.4%), and food-service areas (76.7% to 54.5%). Nevertheless, culture positivity remained persistently high in child-oriented areas (88.9% versus 87.9%) and food-service areas (87.5% versus 90.9%). Food-service samples additionally showed higher priority-microorganism prevalence during the 2022–2023 period (45.5% versus 18.8%), although this comparison involved relatively small denominators. Collectively, descriptive findings indicate lower contamination severity but persistent environmental microbial detection and considerable operational heterogeneity (Table 1).

Annual trajectories showed marked audit-to-audit variation. The low contamination levels observed in 2018 were followed by a pronounced rebound in 2019, while the reduction in high contamination in 2022 was only partly maintained in 2023. Culture positivity remained substantial even when contamination severity was lower, suggesting reduced microbial burden rather than elimination of growth. These patterns varied across functional environments and indicated that pooled period comparisons conceal considerable temporal and operational heterogeneity (Figure 1). The complete ordinal distribution indicated that the reduction in high contamination was accompanied primarily by shifts toward low and moderate contamination rather than a substantial increase in samples without detectable growth (Supplementary Figure S1).

### 3.2. Adjusted Surveillance-Period Associations and Operational Heterogeneity

After adjustment for functional environment and audit-table clustering, culture positivity was similar between the 2022–2023 and pre-COVID periods (adjusted prevalence ratio [aPR], 0.96; 95% CI, 0.83–1.12; *p* = 0.634). In contrast, high contamination was substantially less prevalent during the 2022–2023 period (aPR, 0.36; 95% CI, 0.25–0.54; *p* < 0.001), corresponding to an adjusted absolute reduction of 34.1 percentage points (95% CI, 21.5–46.6). Moderate/high contamination was also lower on the relative scale (aPR, 0.78; 95% CI, 0.61–1.00; *p* = 0.049), although the confidence interval for the adjusted absolute difference included zero. Polymicrobial contamination was less prevalent during the 2022–2023 period (aPR, 0.61; 95% CI, 0.39–0.96; *p* = 0.032), with an adjusted absolute reduction of 8.3 percentage points (95% CI, 1.0–15.7). No corresponding period association was observed for priority-microorganism detection (aPR, 1.06; 95% CI, 0.67–1.67; *p* = 0.804) (Table 2).

High-contamination prevalence ratios were below unity in guest rooms, child-oriented areas, and wellness/water areas; however, evidence of variation across environments was not significant (interaction *p* = 0.150). Priority-microorganism prevalence was descriptively higher in 2022–2023 food-service samples; however, the corresponding estimates were based on limited cluster support, and the overall period-by-environment interaction was inconclusive (*p* = 0.073). Phase comparisons similarly showed markedly lower high contamination in 2022–2023 than in both 2016 and 2018–2019, whereas culture positivity and priority-microorganism prevalence remained broadly unchanged (Figure 2). Lower high-contamination prevalence in 2022–2023 remained consistent across prespecified analytical restrictions, including those addressing sampling-condition comparability, and alternative assumptions regarding missing severity classifications. However, food-service sampling during operation, versus generally post-cleaning sampling elsewhere, limits direct comparisons between environments. Across sensitivity analyses, adjusted prevalence ratios ranged from 0.77–1.15 for culture positivity and 0.85–1.68 for priority-microorganism detection. Leave-one-audit-year-out estimates for culture positivity ranged from 0.86–1.07 (Supplementary Table S1 and Supplementary Figure S2).

### 3.3. Persistent Environmental Surface Hotspots

Among 15 recurring surface categories, eight met the predefined criterion for persistent culture positivity across both surveillance periods. Persistent guest-room hotspots included shower/wet-room floors, bathroom-door handles, light switches, and television remotes. Other handles and controls in child-oriented areas and paddling-pool features in wellness/water areas also remained frequently culture-positive. Food-service hotspots showed high positivity but limited precision: ice-cream machines were positive in 5/6 pre-pandemic samples (83.3%; 95% Wilson CI, 43.6–97.0%) and 6/6 samples in 2022–2023 (100%; 61.0–100%); corresponding estimates for butter machines were 5/6 (83.3%; 43.6–97.0%) and 4/4 (100%; 51.0–100%). Classification was also uncertain near the 50% threshold: bathroom-door handles were positive in 8/14 samples in 2022–2023 (57.1%; 32.6–78.6%). Thus, “persistent hotspot” describes observed positivity rather than establishing that underlying prevalence exceeded 50% in both periods. Priority microorganisms were detected on several persistent surfaces. These findings identify recurring targets for focused environmental-hygiene monitoring; however, persistence refers to surface categories rather than continuous contamination of identical physical objects across audits (Figure 3). A complementary child-focused analysis showed persistently high culture positivity but considerable variation in contamination severity across designated child-oriented areas and harmonized child-use surface classes (Supplementary Figure S3).

### 3.4. Microbiological Profiles and Priority-Organism Distributions

Organism-specific analyses included 538 samples with known microbiological identification status, comprising 262 pre-COVID samples and 276 samples from 2022–2023. Although the overall prevalence of the priority-organism composite was similar between periods, its reported composition differed. The *Bacillus cereus* group decreased from 33/262 samples (12.6%) before COVID to 7/276 (2.5%) during the 2022–2023 period. Conversely, *Staphylococcus aureus* and *Pseudomonas aeruginosa* were each detected in 16/276 samples from 2022–2023 (5.8%) but in no pre-COVID samples. These three period comparisons remained statistically significant after Benjamini–Hochberg adjustment across 13 prespecified organism comparisons (all *q* < 0.001) (Table 3).

*Enterococcus* spp. decreased from 7/262 samples (2.7%) to 1/276 (0.4%), although this comparison did not meet the multiplicity-adjusted significance threshold (*q* = 0.054). *Acinetobacter baumannii*/complex, which was treated as an additional organism of interest rather than a component of the predefined priority-organism composite, decreased from 8/262 samples (3.1%) to 2/276 (0.7%; *q* = 0.083). *Escherichia coli* was detected in two samples from 2022–2023 (0.7%) and in no pre-COVID samples, but the corresponding period comparison was inconclusive (*q* = 0.541). Thus, the similar overall priority-organism composite prevalence masked opposing patterns among its components, particularly reduced *B. cereus* group detection alongside increased *S. aureus* and *P. aeruginosa* detection.

Most *Bacillus cereus* group detections occurred in 2019, whereas *Staphylococcus aureus* and *Pseudomonas aeruginosa* detections were concentrated in 2022. Harmonized organism profiles also differed across functional environments. *Acinetobacter* detection declined markedly in guest rooms and child-oriented areas but remained comparatively frequent in wellness/water settings. *Bacillus* sensu lato increased in child-oriented and food-service areas, while *Staphylococcus* detection remained unchanged in guest rooms but decreased in child-oriented areas (Figure 4).

## 4. Discussion

The present serial surveillance study provides a surface-resolved account of viable bacterial contamination in a family-oriented German hotel across five audits spanning 2016–2023. Three findings define the overall pattern. First, the 2022–2023 period was characterized by a pronounced reduction in high-level contamination, with the adjusted prevalence approximately two-thirds lower than in the pre-COVID period, whereas the probability of recovering any culturable microorganism remained essentially unchanged. Second, polymicrobial recovery decreased, but the overall prevalence of the predefined priority-microorganism composite did not, indicating that reductions in contamination intensity and complexity were not accompanied by an equivalent reduction in all organism-specific indicators. Third, contamination remained strongly structured by functional environment and surface category: child-oriented and food-service areas retained very high culture positivity, several recurring touch or wet surfaces remained persistent hotspots, and the organisms contributing to the priority composite changed substantially over time. Taken together, these findings show that environmental hygiene cannot be adequately represented by a single positive/negative endpoint. The hotel’s later surveillance profile was more consistent with lower bacterial burden and less frequent polymicrobial recovery than with broad microbiological elimination.

The divergence between culture positivity and colony burden is central to interpretation. A culture-positive result indicates that viable, culturable organisms were recoverable under the applied laboratory conditions, but it does not distinguish sparse deposition from dense growth, nor does it establish transmission potential or clinical risk. Culture-based investigations also capture only the fraction of the environmental microbiota able to grow on the selected media under the specified incubation conditions [6,37]. Accordingly, the stable prevalence of culture positivity alongside a marked decline in high contamination is biologically plausible: cleaning may remove a large proportion of surface biomass while leaving residual organisms, and frequently contacted surfaces may be rapidly re-seeded by occupants or staff [9]. A healthcare-bioburden study similarly found that more intensive terminal cleaning reduced mean colony counts and multidrug-resistant-organism recovery without rendering all surfaces culture-negative [21]. In a hotel-lobby intervention, targeted hygiene produced a large reduction in tracer concentration and cross-contamination, but a smaller change in the proportion of positive surfaces, further illustrating that contamination magnitude and detection frequency need not move in parallel [7]. The present results therefore support reporting at least culture positivity, contamination severity, and polymicrobial recovery together; reliance on culture positivity alone would have obscured the most robust temporal change observed in this hotel.

The lower prevalence of high and polymicrobial contamination during 2022–2023 is compatible with several explanations, including changes in hygiene practices, occupancy, and environmental conditions; however, these explanations remain hypotheses because the relevant covariates were not measured. Across the hospitality sector, the pandemic increased the salience of cleanliness, expanded disinfection and hand-hygiene practices, and prompted changes to housekeeping, staff procedures, technology, and shared-space management [24,25]. Such changes could preferentially reduce accumulated biomass and interrupt repeated transfer between hands and surfaces, even when ongoing occupancy continues to introduce microorganisms. Targeted intervention work in a functioning hotel supports this mechanism by showing that strategically treating high-contact sites can reduce contamination even at some untreated sites, presumably by disrupting transfer pathways [7]. However, the present surveillance design cannot attribute the observed period contrast to a particular intervention, or to pandemic-related hygiene alone. Epidemics can markedly alter hotel occupancy, facility use, and operating decisions, all of which may change the intensity and spatial distribution of microbial deposition [28]. The irregular annual trajectory (especially the low levels in 2018, rebound in 2019, strong decrease in 2022, and partial rebound in 2023) also argues against interpreting the data as a simple monotonic improvement. The comparison between the pre-pandemic period and 2022–2023 therefore represents a temporal contrast rather than an estimate of the causal effect of pandemic-related hygiene measures. Robustness analyses consistently supported lower high contamination across alternative restrictions, assumptions, clustering approaches, and individual audit-year exclusions. Culture positivity and priority-microorganism estimates were less stable. Thus, evidence was strongest for reduced contamination intensity during the 2022–2023 period, but weaker for changes in overall microbial detectability or priority-microorganism presence.

Functional-area heterogeneity provides a second major insight. Indoor microbial communities are shaped jointly by occupants, contact patterns, surface type, moisture, ventilation, and local environmental conditions; dry touch surfaces and wet installations consequently form distinct ecological niches rather than interchangeable sampling units [4,23]. In the present study, high contamination decreased in every functional environment, suggesting a hotel-wide shift in burden. Nevertheless, the residual profile was uneven. Guest rooms showed the largest absolute improvement and a low prevalence of high contamination during 2022–2023, whereas child-oriented and food-service areas remained culture-positive in approximately nine out of ten samples. Food-service equipment also retained the greatest prevalence of high contamination in the later period. Although the formal interaction test did not establish statistically significant between-environment variation for high contamination, the descriptive pattern identifies operational zones in which exposure opportunities, surface design, moisture, and replenishment of organic material may counteract otherwise effective routine hygiene.

The child-oriented findings are consistent with the particular ecology of shared play environments. Childcare settings are characterized by dense occupancy, frequent contact with floors, toys, handles, and other shared objects, and substantial dispersal of human-associated bacteria across high-use surfaces [13]. Children’s greater hand-to-surface and hand-to-mouth activity can also increase multipath exposure opportunities relative to adults [14,38]. In this hotel, the persistence of culture positivity on child-area handles and controls, despite lower high-level contamination overall, suggests rapid re-deposition onto intensively shared surfaces or incomplete access during cleaning. This pattern does not demonstrate elevated infection risk: studies in communal residential settings have shown that prevalent surface contamination may be poorly correlated with reported illness, and the clinical significance of environmental detection depends on organism identity, dose, transfer efficiency, host susceptibility, and behavior [12,39]. It does, however, support short-interval, use-responsive hygiene for a limited set of high-contact child-use surfaces rather than treating the entire area as microbiologically uniform.

The food-service results require similarly differentiated interpretation. Food-processing environments commonly contain recurring communities that include *Pseudomonas*, *Acinetobacter*, *Staphylococcus*, and *Bacillus*, with assembly influenced by the food commodity, water, nutrients, equipment architecture, and sanitation regime [17,19]. Joints, dispensers, internal contact surfaces, and other difficult-to-access components can retain residues and support attachment or biofilm formation, allowing localized contamination to recur even when exposed surfaces are cleaned [40,41,42,43]. The persistent positivity of ice-cream and butter machines, together with the high residual burden in the food-service stratum, is therefore operationally important. It suggests that verification should extend beyond cleaning frequency to equipment-specific disassembly, access, drying, sanitation compatibility, and post-cleaning microbiological checks. Observations from food-service units also identify food handlers’ hands, utensils, and inadequately sanitized contact surfaces as potential links in contamination pathways [19]. Nevertheless, the apparent increase in priority-microorganism detection in the 2022–2023 food-service samples was based on small denominators and an inconclusive interaction test; it should be treated as a signal for targeted follow-up rather than evidence of a hotel-wide deterioration.

The wellness and water-associated findings point to a different mechanism. Water-related indoor habitats select microorganisms able to tolerate moisture, fluctuating nutrients, disinfectant stress, and surface-associated growth, and plumbing or bathing installations may sustain biofilms that repeatedly seed adjacent surfaces [15,44,45,46]. Material properties, temperature, relative humidity, inoculum size, and organism identity further affect survival on inanimate surfaces [1,5,47,48]. These mechanisms are consistent with continued positivity on paddling-pool features and with the comparatively frequent recovery of *Acinetobacter* in wellness/water samples, even as *Acinetobacter* declined in guest rooms and child-oriented areas. They also imply that repeated culture positivity in a wet niche should not automatically be interpreted as failure of routine wiping; it may instead indicate recolonization from a protected reservoir. Evaluation of such sites should therefore include drainage, water flow, drying, surface integrity, and biofilm-prone interfaces, with corrective action tailored to the installation rather than extrapolated from dry high-touch surfaces.

The hotspot analysis identified recurrent operational vulnerabilities, including wet-room floors, frequently touched controls, paddling-pool features, and food-service machinery. Their persistence likely reflects different mechanisms, including repeated hand-mediated deposition, moisture-associated recolonization, and limited cleaning access; accordingly, surveillance and hygiene measures should be tailored to surface function and risk [7,10]. Because recurrence was demonstrated for harmonized surface categories rather than identical physical objects, these findings should not be interpreted as continuous contamination of individual items.

Although the overall priority-microorganism prevalence remained unchanged, its composition shifted: the *B. cereus* group declined, *S. aureus* and *P. aeruginosa* were detected only during the 2022–2023 period, and *Enterococcus* and *A. baumannii*/complex tended to decrease. The composite therefore masked opposing organism-specific patterns and should not be interpreted as evidence of an unchanged microbiological profile. Most *B. cereus* group detections occurred in 2019, whereas *S. aureus* and *P. aeruginosa* detections clustered in 2022, suggesting substantial audit-specific rather than progressive temporal variation. Accordingly, the pooled pre-COVID versus 2022–2023 contrasts for these organisms should not be interpreted as evidence of a stable increase throughout the later surveillance period. Their ecological distributions are compatible with dust, food, human-contact, and moisture-associated sources [1,15,17,41,49,50]; however, sampling and reporting differences may have influenced recovery. Community carriage of *S. aureus* among adults in Germany further supports hotel occupants and staff as plausible sources of environmental introduction, although the present data cannot attribute individual isolates to a specific source [51]. Without strain characterization or clinical linkage, these findings support confirmatory surveillance but do not establish microbial emergence, transmission, or health risk.

Several practical implications follow. First, environmental-hygiene performance should be assessed with a small panel of complementary measures: positivity for detecting the breadth of viable contamination, ordinal or quantitative burden for identifying substantial accumulation, polymicrobial recovery for contamination complexity, and organism-specific indicators for hygienic relevance. Second, audit design should retain core sentinel surfaces across years while adding function-specific targets in response to changing operations. Third, hygiene interventions should prioritize recurrent transfer nodes and difficult-to-clean equipment rather than indiscriminately increasing chemical application across all surfaces. A hotel-specific targeted-hygiene study supports prioritizing the right surfaces and moments as an effective and more resource-conscious approach [7]. Finally, persistent wet and food-associated sites require engineering and process controls—such as equipment access, drainage, drying, maintenance, and validated disassembly procedures—in addition to routine wiping. Food-safety research likewise emphasizes that a positive culture is not invariably evidence of system failure and that a reduction in counts does not by itself guarantee control; interpretation must be embedded in longitudinal, process-aware monitoring [40].

Several limitations define the scope of inference. The data came from one family-oriented hotel in Bavaria, and the results may not generalize to other German hotels with different architecture, clientele, occupancy, housekeeping systems, water installations, food operations, or climate. The audits were serial cross-sectional assessments conducted at irregular intervals, with no reports available for 2020–2021; they cannot establish a continuous temporal trend or the immediate effect of pandemic restrictions. Although sampling area and swabbing pressure were standardized across audits, the physical objects sampled within recurring surface categories were not necessarily identical. Differences in surface composition and sampling conditions may therefore have influenced CFU-based severity comparisons. Occupancy, contact frequency, time since cleaning, cleaning product formulation, changes in disinfection protocols, staff adherence, temperature, relative humidity, ventilation, water characteristics, and equipment maintenance were not available for direct adjustment. Differences in the sampled surfaces and sampling conditions across audits may have produced residual confounding despite common-support and recurring-surface analyses. In addition, airborne culturable bacteria and outdoor-to-indoor microbial infiltration were not assessed, preventing evaluation of their contributions to surface contamination. Culture and MALDI-TOF identification provided evidence about viable, recoverable organisms and useful taxonomic resolution, but did not characterize bacteria not recoverable under the applied culture conditions, total community structure, resistance genes, virulence determinants, or strain relatedness [6,37,52]. Without antimicrobial susceptibility testing, resistance phenotypes, including methicillin resistance in *S. aureus*, could not be determined. Detection of priority microorganisms therefore indicates potential hygienic relevance but does not establish contamination with resistant strains or quantify infection risk. Some environment-specific and organism-specific comparisons had small denominators or few clusters, limiting precision; organism-specific tests also lacked adjustment for functional environment and audit clustering. Unadjusted outcome and phase comparisons carry an increased false-positive risk, particularly for the borderline findings on moderate/high and polymicrobial contamination. Finally, repeated positivity was established at the harmonized surface-category level, not through longitudinal tracking of identical objects, and no health outcomes were measured; the study therefore characterizes environmental hygiene rather than infection risk.

Despite these limitations, the present study has several strengths. It evaluated a substantial surface-level dataset from one hotel across five audits and more than seven years, permitting within-setting comparison that is understudied in environmental microbiology. The analysis integrated burden, positivity, polymicrobial recovery, priority microorganisms, functional environments, recurring surfaces, and organism profiles rather than relying on a single endpoint. It also used cluster-robust adjusted models, multiplicity control for organism-specific comparisons, and extensive sensitivity analyses addressing missingness, sampling comparability, surface support, audit composition, and clustering. The convergence of these analyses for high contamination supports the internal robustness of the principal finding, while the less stable results for other outcomes were transparently retained rather than overinterpreted. Future studies should combine standardized longitudinal sampling with contemporaneous operational and environmental data to distinguish incomplete cleaning from rapid recontamination at persistent hotspots [53]. Multicenter intervention studies integrating culture with molecular and isolate-level analyses are needed to assess generalizability and determine whether surface-specific controls, including adjunctive no-touch disinfection technologies such as hydrogen peroxide nebulization [54], produce sustained reductions in bacterial burden and delay recolonization relative to routine cleaning. Such evaluations should also address application parameters, material compatibility, occupational safety, and operational feasibility in hospitality settings.

## 5. Conclusions

Serial surveillance of the investigated German hotel demonstrated an adjusted 34.1-percentage-point reduction in high-level bacterial contamination during the 2022–2023 period (95% CI, 21.5–46.6), together with less frequent polymicrobial recovery, despite no material change in culture positivity or aggregate priority-microorganism prevalence. Persistent hotspots, functional-area heterogeneity, and shifts in organism composition demonstrate that lower overall burden does not eliminate localized hygiene vulnerabilities. These findings support longitudinal, risk-oriented hospitality hygiene that integrates contamination severity, surface function, ecological context, and organism identity to prioritize cleaning, disinfection, maintenance, and microbiological verification.

## Figures and Tables

**Figure 1:**
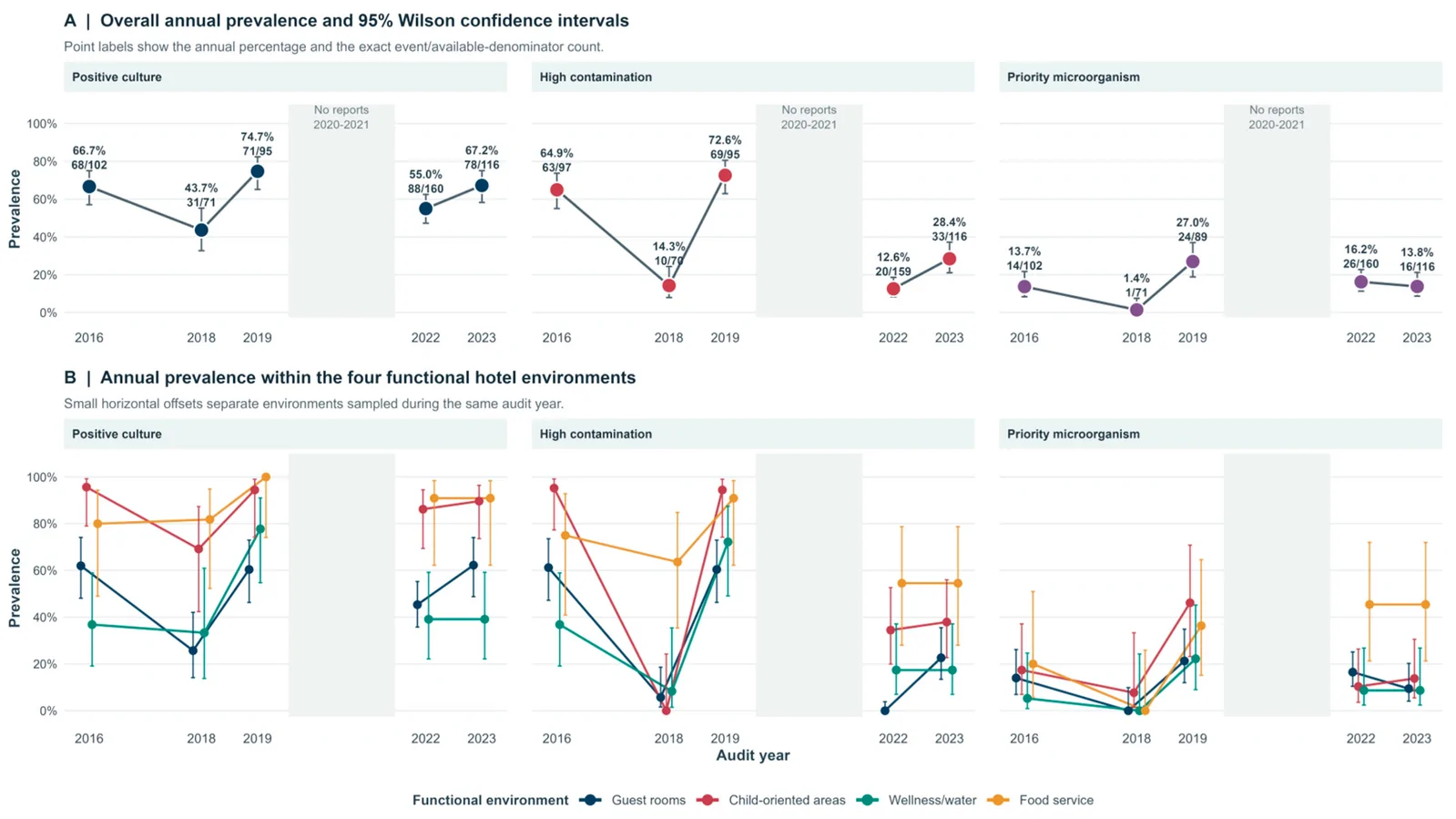
Audit-year trajectories of environmental microbiological outcomes overall and by functional environment. Panel (**A**) shows overall prevalence with 95% Wilson confidence intervals; Panel (**B**) shows prevalence by functional environment. Labels give events/available denominator. High contamination was defined as >500 CFU or confluent growth.

**Figure 2:**
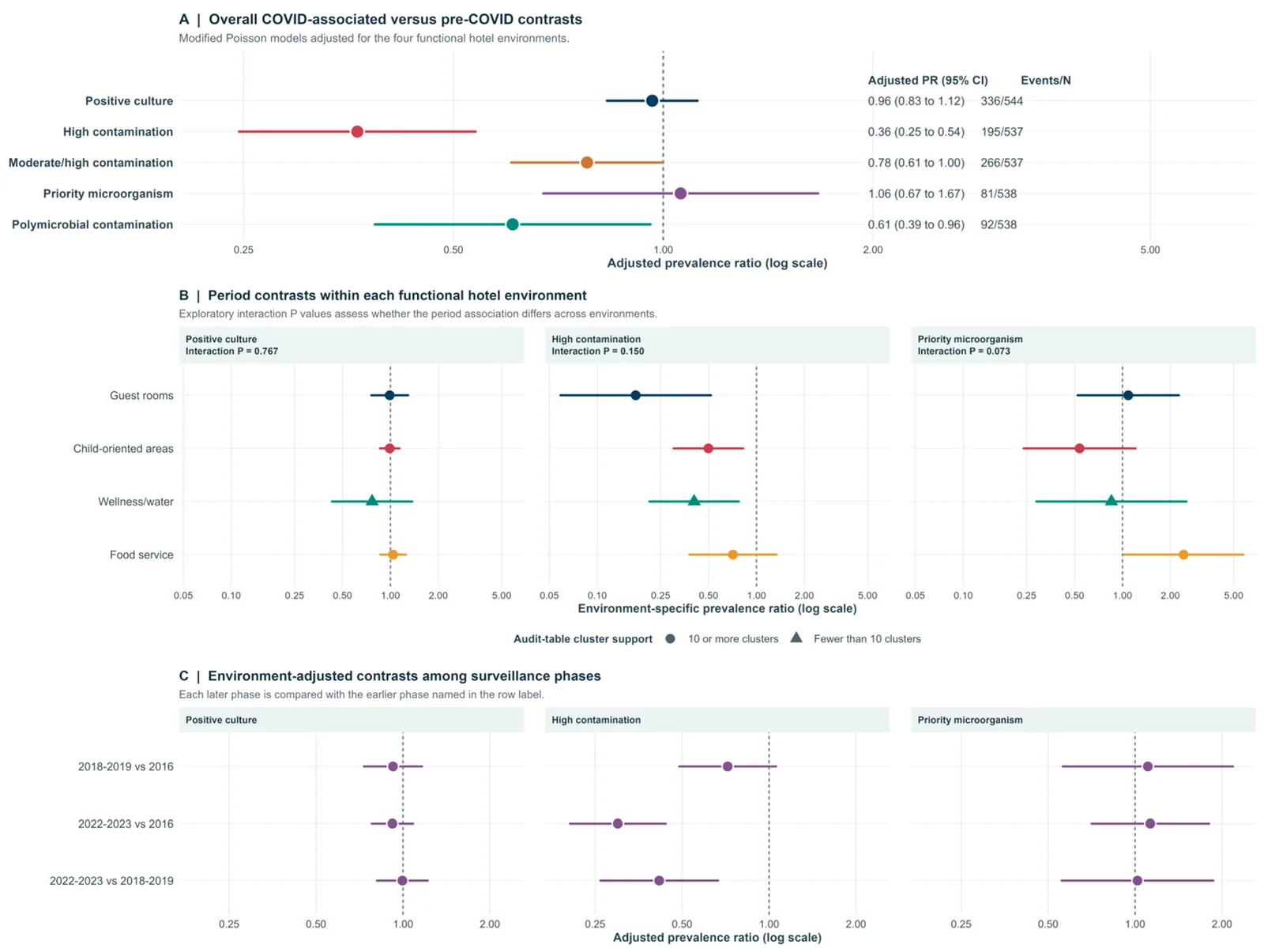
Adjusted prevalence-ratio estimates across surveillance periods, functional environments, and surveillance phases. Points represent prevalence ratios (PRs), and horizontal lines represent 95% HC1 cluster-robust confidence intervals; dashed vertical lines indicate PR = 1. Panel (**A**) presents 2022–2023 versus pre-COVID estimates adjusted for functional environment. Panel (**B**) presents period contrasts within each environment and global interaction *p*-values. Panel (**C**) presents environment-adjusted contrasts between surveillance phases. Clusters were defined by audit year and source-report table; triangles indicate estimates based on fewer than 10 clusters. Model-based *p*-values and confidence intervals are not adjusted for multiple comparisons.

**Figure 3:**
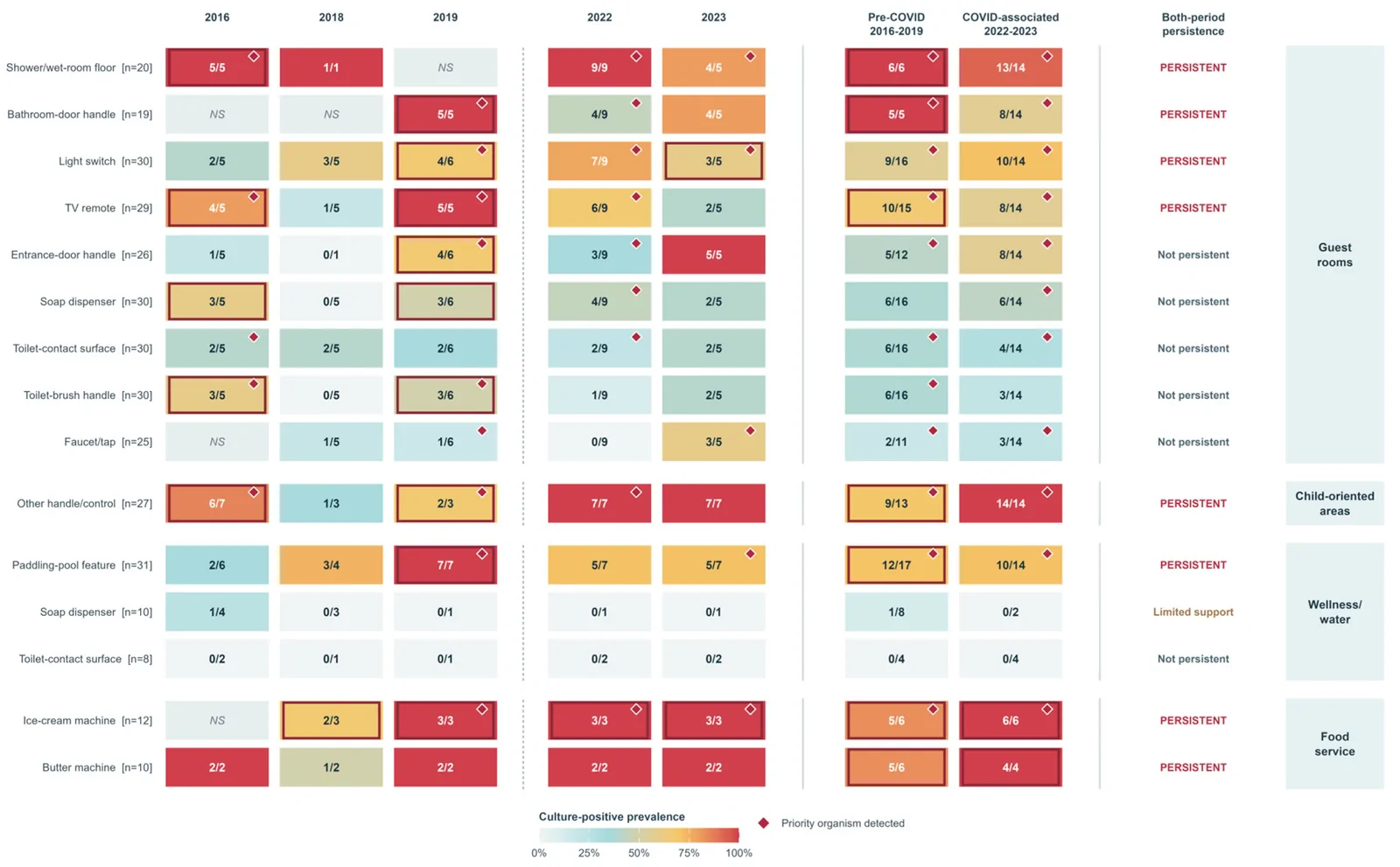
Persistent environmental surface hotspots across five hotel-hygiene audits. Tile colors represent culture-positive prevalence, and labels show positive/available samples; NS indicates not sampled. Diamonds indicate detection of at least one priority microorganism. Dark-red outlines identify cells in which at least 50% of evaluable samples had high contamination, based on at least three samples. Eligible categories had at least eight samples overall and were represented in at least three audit years. Persistence was defined as at least 50% culture positivity in both pooled periods, with at least three samples per period.

**Figure 4:**
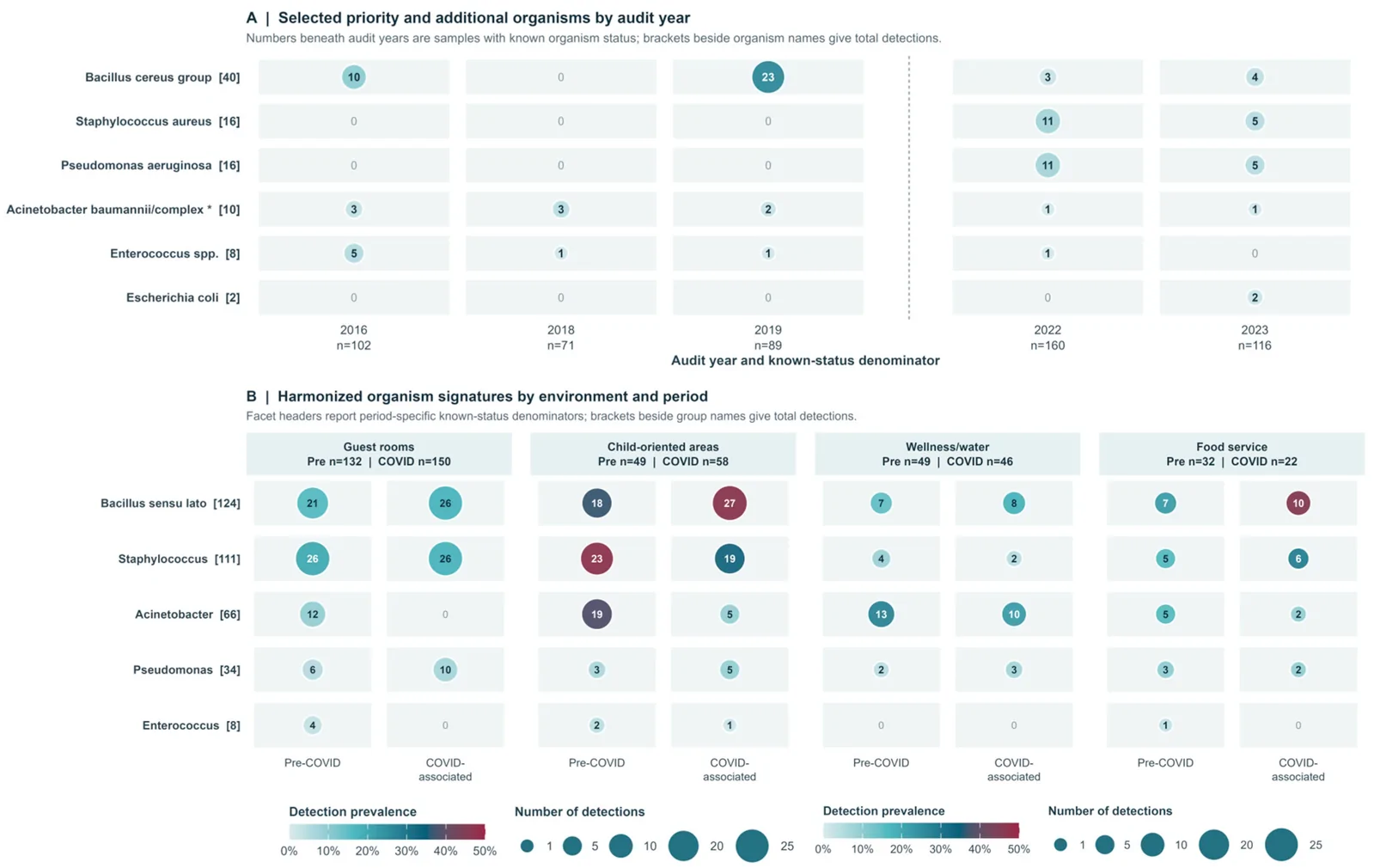
Temporal and environment-specific microbiological signatures across surveillance audits and periods. Bubble size and printed values indicate the number of detections; color represents prevalence among samples with known organism status. Panel (**A**) presents selected organisms by audit year, and Panel (**B**) presents harmonized organism groups by functional environment and surveillance period. Culture-negative samples were classified as organism-negative; six unidentified positive cultures from 2019 were excluded. * *A. baumannii*/complex was an additional organism of interest and was not included in the predefined priority composite. *Bacillus* sensu lato combines reported *Bacillus*, *Priestia*, and *Lysinibacillus* labels for analytical harmonization.

**Table 1:** Environmental microbiological contamination outcomes across audit years and surveillance phases, stratified by functional environment.

Year/Phase	Sample	Positive Culture	High Contamination	Priority Microorganism	Polymicrobial Contamination	Fecal Indicator
Pre-COVID Initial audit (2016)	102	68 (66.7%)	63/97 (64.9%)	14/102 (13.7%)	31/102 (30.4%)	5/102 (4.9%)
Pre-COVID follow-up (2018–2019)	166	102 (61.4%)	79/165 (47.9%)	25/160 (15.6%)	26/160 (16.2%)	2/160 (1.2%)
Total pre-COVID phase	268	170 (63.4%)	142/262 (54.2%)	39/262 (14.9%)	57/262 (21.8%)	7/262 (2.7%)
*Guest rooms*	133	69 (51.9%)	61/132 (46.2%)	17/132 (12.9%)	14/132 (10.6%)	4/132 (3.0%)
*Child-oriented areas*	54	48 (88.9%)	37/51 (72.5%)	11/49 (22.4%)	28/49 (57.1%)	2/49 (4.1%)
*Wellness/water*	49	25 (51.0%)	21/49 (42.9%)	5/49 (10.2%)	4/49 (8.2%)	0/49 (0.0%)
*Food service*	32	28 (87.5%)	23/30 (76.7%)	6/32 (18.8%)	11/32 (34.4%)	1/32 (3.1%)
*Total 2018*	71	31 (43.7%)	10/70 (14.3%)	1/71 (1.4%)	6/71 (8.5%)	1/71 (1.4%)
*Total 2019*	95	71 (74.7%)	69/95 (72.6%)	24/89 (27.0%)	20/89 (22.5%)	1/89 (1.1%)
2022–2023 phase	276	166 (60.1%)	53/275 (19.3%)	42/276 (15.2%)	35/276 (12.7%)	3/276 (1.1%)
*Guest rooms*	150	77 (51.3%)	12/149 (8.1%)	21/150 (14.0%)	8/150 (5.3%)	0/150 (0.0%)
*Child-oriented areas*	58	51 (87.9%)	21/58 (36.2%)	7/58 (12.1%)	11/58 (19.0%)	3/58 (5.2%)
*Wellness/water*	46	18 (39.1%)	8/46 (17.4%)	4/46 (8.7%)	4/46 (8.7%)	0/46 (0.0%)
*Food service*	22	20 (90.9%)	12/22 (54.5%)	10/22 (45.5%)	12/22 (54.5%)	0/22 (0.0%)
*Total 2022*	160	88 (55.0%)	20/159 (12.6%)	26/160 (16.2%)	16/160 (10.0%)	1/160 (0.6%)
*Total 2023*	116	78 (67.2%)	33/116 (28.4%)	16/116 (13.8%)	19/116 (16.4%)	2/116 (1.7%)

Culture positivity was defined as any detectable microbial growth. High contamination was defined as more than 500 CFU or confluent growth. Priority-microorganism detection was defined as the presence of at least one predefined priority microorganism; polymicrobial contamination as the detection of at least two distinct standardized microorganism taxa in the same sample; and fecal-indicator detection as the presence of *Enterococcus* spp. or *Escherichia coli*. Denominators are outcome-specific: high contamination uses samples with evaluable severity (pre-pandemic, n = 262; 2022–2023, n = 275), whereas priority-microorganism detection uses samples with known organism status (n = 262 and n = 276, respectively). Identical denominator counts do not necessarily indicate identical sample sets.

**Table 2:** Adjusted associations between surveillance period and environmental microbiological outcomes.

Outcome	Events/N	Adjusted PR (95% CI)	Adjusted Difference, Percentage Points (95% CI)	*p*-Value	Period–Environment Interaction *p*-Value
Positive culture	336/544	0.96 (0.83 to 1.12)	−2.2 (−11.5 to 7.1)	0.634	0.767
High contamination	195/537	0.36 (0.25 to 0.54)	−34.1 (−46.6 to −21.5)	<0.001	0.150
Moderate/high contamination	266/537	0.78 (0.61 to 1.00)	−12.4 (−25.1 to 0.3)	0.049	0.333
Priority microorganism	81/538	1.06 (0.67 to 1.67)	0.9 (−5.9 to 7.6)	0.804	0.073
Polymicrobial contamination	92/538	0.61 (0.39 to 0.96)	−8.3 (−15.7 to −1.0)	0.032	0.037

PR, prevalence ratio; CI, confidence interval. Values in Events/N are events divided by the outcome-specific available denominator. Adjusted PRs were estimated using modified Poisson regression with a log link and adjustment for functional environment. HC1 cluster-robust covariance was calculated across 57 audit-year-by-source-table clusters. Adjusted differences are model-standardized 2022–2023 minus pre-COVID prevalence differences. Interaction *p*-values are joint cluster-robust Wald tests of all period-by-environment terms. Model-based *p*-values and confidence intervals are not adjusted for multiple comparisons.

**Table 3:** Prevalence of selected priority and additional microorganisms by surveillance period.

Taxon	Pre-COVID, n/N (%)	2022–2023, n/N (%)	Absolute Difference, Percentage Points	BH-Adjusted *q*-Value
*Bacillus cereus* group	33/262 (12.6%)	7/276 (2.5%)	−10.1	<0.001
*Staphylococcus aureus*	0/262 (0.0%)	16/276 (5.8%)	+5.8	<0.001
*Pseudomonas aeruginosa*	0/262 (0.0%)	16/276 (5.8%)	+5.8	<0.001
*Acinetobacter baumannii*/complex	8/262 (3.1%)	2/276 (0.7%)	−2.3	0.083
*Enterococcus* spp.	7/262 (2.7%)	1/276 (0.4%)	−2.3	0.054
*Escherichia coli*	0/262 (0.0%)	2/276 (0.7%)	+0.7	0.541

Values represent detections divided by samples with known organism status (%). Absolute differences are 2022–2023 minus pre-COVID prevalence in percentage points. BH, Benjamini–Hochberg. The predefined priority-organism composite comprises the *Bacillus cereus* group, *Staphylococcus aureus*, *Pseudomonas aeruginosa*, *Enterococcus* spp., and *Escherichia coli*. *Acinetobacter baumannii*/complex was an additional organism of interest and was not part of this composite. Two-sided Fisher exact tests were exploratory and unadjusted for environment or audit-table clustering. Reported *q*-values control the false-discovery rate across all 13 prespecified organism comparisons conducted in the analysis.

## Data Availability

The original contributions presented in this study are included in the article. Further inquiries can be directed to the corresponding authors.

## Supplementary Materials

The following supporting information can be downloaded at https://www.mdpi.com/article/10.3390/microorganisms14102183/s1, Table S1: Sensitivity, sampling comparability, and audit-year influence analyses of surveillance-period contrasts; Figure S1: Complete ordinal contamination-severity distributions across audit years, surveillance periods, and functional environments; Figure S2: Sensitivity analyses, sampling comparability, and audit-year influence on surveillance-period associations; Figure S3: Environmental hygiene outcomes in child-oriented areas and across child-use surface classes.

## Abbreviations

The following abbreviations are used in this manuscript:

16S rRNA	16S ribosomal RNA
aPR	adjusted prevalence ratio
BH	Benjamini–Hochberg
CFU	colony-forming unit
CI	confidence interval
COVID-19	coronavirus disease 2019
HC1	heteroskedasticity-consistent type 1 covariance estimator
MALDI-TOF MS	matrix-assisted laser desorption/ionization time-of-flight mass spectrometry
PR	prevalence ratio
SARS-CoV-2	severe acute respiratory syndrome coronavirus 2

## References

[1] Wißmann J.E., Kirchhoff L., Brüggemann Y., Todt D., Steinmann J., Steinmann E. (2021). Persistence of pathogens on inanimate surfaces: A narrative review. Microorganisms.

[2] Siwek M., Kolasińska A., Wrześniewski K., Zmuda-Pałka M. (2022). Services and amenities offered by city hotels within family tourism as one of the factors guaranteeing satisfactory leisure time. Int. J. Environ. Res. Public Health.

[3] Almanza B.A., Kirsch K., Kline S.F., Sirsat S., Stroia O., Choi J.K., Neal J. (2015). How clean are hotel rooms? Part I: Visual observations vs. microbiological contamination. J. Environ. Health.

[4] Hou J., Nakajima M., Nishiuchi Y., Ogura D., Teramoto A., Kuratomi C., Iwamoto Y., Okamura Y., Moriguchi K., Dovjak M. (2025). Occupants and surface types drive microbial dynamics in controlled indoor environments. Environ. Microbiome.

[5] Cheng H., Chen J., Yu H., Sun B., Zhou J., Wu G. (2025). Environmental factors and antimicrobial efficacy: The impact of temperature and humidity on material surfaces. Microbiol. Spectr..

[6] Chawla H., Anand P., Garg K., Bhagat N., Varmani S.G., Bansal T., McBain A.J., Marwah R.G. (2023). A comprehensive review of microbial contamination in the indoor environment: Sources, sampling, health risks, and mitigation strategies. Front. Public Health.

[7] Spitzer M.E., Jung Y., Sexton J.D., Wilson A.M., Picton J.L., Miura-Akagi B.T., Buckley C., Upson S.E., Ackerley L.M., Gent L. (2025). Quantifying fomite hotspots and targeted hygiene impacts in a hotel lobby. Int. J. Hyg. Environ. Health.

[8] Fu X., Li Y., Yuan Q., Cai G.H., Deng Y., Zhang X., Norbäck D., Sun Y. (2020). Continental-scale microbiome study reveals different environmental characteristics determining microbial richness, composition, and quantity in hotel rooms. mSystems.

[9] Lax S., Smith D.P., Hampton-Marcell J., Owens S.M., Handley K.M., Scott N.M., Gibbons S.M., Larsen P., Shogan B.D., Weiss S. (2014). Longitudinal analysis of microbial interaction between humans and the indoor environment. Science.

[10] Martens A., Schauer M., Mair S., Motevalli M., König B. (2026). Multidimensional Profiles of Microbial Contamination and Hygiene Risk Across Functional Areas in Family Hotels. Life.

[11] Cao H., Ye H., Tian Y., Zhang J., Xie Y., Chen Y., Mo Q., Huang S., Tao Y., Liu T. (2025). Functional Areas Shape Indoor Microbial Structure and Potential Risks in University Dormitories. Front. Microbiol..

[12] Miko B.A., Cohen B., Haxall K., Conway L., Kelly N., Stare D., Tropiano C., Gilman A., Seward S.L., Larson E. (2013). Personal and household hygiene, environmental contamination, and health in undergraduate residence halls in New York City, 2011. PLoS ONE.

[13] Beasley D.E., Monsur M., Hu J., Dunn R.R., Madden A.A., Martin L.J. (2022). The bacterial community of childcare centers: Potential implications for microbial dispersal and child exposure. Environ. Microbiome.

[14] Ferguson A., Penney R., Solo-Gabriele H. (2017). A review of the field on children’s exposure to environmental contaminants: A risk assessment approach. Int. J. Environ. Res. Public Health.

[15] Novak Babič M., Gostinčar C., Gunde-Cimerman N. (2020). Microorganisms populating the water-related indoor biome. Appl. Microbiol. Biotechnol..

[16] Lagière J., Labarthe S., Dubourg K., Bauduer F. (2020). Influence of hydrotherapy pool water recirculation regime on *Staphylococcus* species concentration at subsurface: Preliminary experimental data from a pilot study. Environ. Int..

[17] Xu Z.S., Ju T., Yang X., Gänzle M. (2023). A meta-analysis of bacterial communities in food processing facilities: Driving forces for assembly of core and accessory microbiomes across different food commodities. Microorganisms.

[18] Lebreton M., Michel-Le Roux S., Lagarde J., La T.P., Brothier E., Nazaret S., Abrouk D., Sarrazin M., Séclet-Ziebal C., Pourcher A.M. (2026). Food waste as a vector of foodborne and opportunistic pathogens: A study on microbial contamination in an urban area. Waste Manag..

[19] Alves A., Viveiros C., Lopes J., Nogueira A., Pires B., Afonso A.F., Teixeira C. (2021). Microbiological contamination in different food service units associated with food handling. Appl. Sci..

[20] Dancer S.J. (2004). How do we assess hospital cleaning? A proposal for microbiological standards for surface hygiene in hospitals. J. Hosp. Infect..

[21] Shams A.M., Rose L.J., Edwards J.R., Cali S., Harris A.D., Jacob J.T., LaFae A., Pineles L.L., Thom K.A., McDonald L.C. (2016). Assessment of the Overall and Multidrug-Resistant Organism Bioburden on Environmental Surfaces in Healthcare Facilities. Infect. Control Hosp. Epidemiol..

[22] Yang J., Qin X., Zhang D., Dong C. (2026). Microbial landscape: Composition and health associations of environmental microbiome in key functional spaces of premium elderly care facilities. Microbiol. Spectr..

[23] Gilbert J.A., Stephens B. (2018). Microbiology of the built environment. Nat. Rev. Microbiol..

[24] Pillai S.G., Haldorai K., Seo W.S., Kim W.G. (2021). COVID-19 and hospitality 5.0: Redefining hospitality operations. Int. J. Hosp. Manag..

[25] Bonfanti A., Vigolo V., Yfantidou G. (2021). The impact of the COVID-19 pandemic on customer experience design: The hotel managers’ perspective. Int. J. Hosp. Manag..

[26] Klassert T.E., Zubiria-Barrera C., Neubert R., Stock M., Schneegans A., López M., Driesch D., Zakonsky G., Gastmeier P., Slevogt H. (2022). Comparative analysis of surface sanitization protocols on the bacterial community structures in the hospital environment. Clin. Microbiol. Infect..

[27] Martens A., Schauer M., Motevalli M., Mair S., König B. (2026). Microbial Contamination of Gym Equipment: Diversity Patterns, Temporal Dynamics, Staphylococcus Hotspots, and Device-Level Risk Indices. Pathogens.

[28] Wu E.H.C., Law R., Jiang B. (2010). The impact of infectious diseases on hotel occupancy rate based on independent component analysis. Int. J. Hosp. Manag..

[29] Bedrosian N., Mitchell E., Rohm E., Rothe M., Kelly C., String G., Lantagne D. (2021). A Systematic Review of Surface Contamination, Stability, and Disinfection Data on SARS-CoV-2 (Through July 10, 2020). Environ. Sci. Technol..

[30] Gonçalves J., da Silva P.G., Reis L., Nascimento M.S.J., Koritnik T., Paragi M., Mesquita J.R. (2021). Surface contamination with SARS-CoV-2: A systematic review. Sci. Total Environ..

[31] Copan Italia S.p.A. Copan Liquid Amies Elution Swab (ESwab^®^) Collection and Transport System: Product Insert & How to Use Guide; HPC108 Rev.00; July 2017. https://www.copanusa.com/wp-content/uploads/2020/11/ESwab-Package-Insert.pdf?utm_source.

[32] Babiker A., Van Riel J., Lohsen S., Page A., Strudwick A., Wilber E., Woodworth M., Satola S. (2025). Evaluation of Swabbing Methods for Culture and Non-Culture-Based Recovery of Multidrug-Resistant Organisms from Environmental Surfaces. Infect. Control Hosp. Epidemiol..

[33] Dubois D., Grare M., Prere M.F., Segonds C., Marty N., Oswald E. (2012). Performances of the Vitek MS Matrix-Assisted Laser Desorption Ionization–Time of Flight Mass Spectrometry System for Rapid Identification of Bacteria in Routine Clinical Microbiology. J. Clin. Microbiol..

[34] Rychert J., Burnham C.A., Bythrow M., Garner O.B., Ginocchio C.C., Jennemann R., Lewinski M.A., Manji R., Mochon A.B., Procop G.W. (2013). Multicenter Evaluation of the Vitek MS Matrix-Assisted Laser Desorption Ionization–Time of Flight Mass Spectrometry System for Identification of Gram-Positive Aerobic Bacteria. J. Clin. Microbiol..

[35] Patra J.K., Das G., Das S.K., Thatoi H. (2020). Isolation, culture, and biochemical characterization of microbes. A Practical Guide to Environmental Biotechnology.

[36] (2022). Medical Laboratories—Requirements for Quality and Competence.

[37] Knight R., Vrbanac A., Taylor B.C., Aksenov A., Callewaert C., Debelius J., Gonzalez A., Kosciolek T., McCall L.I., McDonald D. (2018). Best practices for analysing microbiomes. Nat. Rev. Microbiol..

[38] Liu Z., Hu L., Hu C., Liu Q., Liang Z., Rong R., Liu H. (2023). Aggravated exposure risks of children to multipath transmitted pathogens in indoor environments. iScience.

[39] Lopez G.U., Gerba C.P., Tamimi A.H., Kitajima M., Maxwell S.L., Rose J.B. (2013). Transfer efficiency of bacteria and viruses from porous and nonporous fomites to fingers under different relative humidity conditions. Appl. Environ. Microbiol..

[40] Omelchenko O., Diaz M., Gutiérrez A.V., Webber M.A., Wilson N., Gilmour M. (2026). Food safety culture and the control of microbial communities in food production environments. npj Antimicrob. Resist..

[41] Liu N.T., Bauchan G.R., Francoeur C.B., Shelton D.R., Lo Y.M., Nou X. (2016). *Ralstonia insidiosa* serves as a bridge in biofilm formation by foodborne pathogens *Listeria monocytogenes*, *Salmonella enterica*, and enterohemorrhagic *Escherichia coli*. Food Control.

[42] Abdallah M., Benoliel C., Drider D., Dhulster P., Chihib N.E. (2014). Biofilm formation and persistence on abiotic surfaces in the context of food and medical environments. Arch. Microbiol..

[43] Tirpanci Sivri G., Abdelhamid A.G., Kasler D.R., Yousef A.E. (2023). Removal of *Pseudomonas fluorescens* biofilms from pilot-scale food processing equipment using ozone-assisted cleaning-in-place. Front. Microbiol..

[44] Yano T., Kubota H., Hanai J., Hitomi J., Tokuda H. (2013). Stress tolerance of *Methylobacterium* biofilms in bathrooms. Microbes Environ..

[45] De Sotto R., Tang R., Bae S. (2020). Biofilms in premise plumbing systems as a double-edged sword: Microbial community composition and functional profiling of biofilms in a tropical region. J. Water Health.

[46] Feazel L.M., Baumgartner L.K., Peterson K.L., Frank D.N., Harris J.K., Pace N.R. (2009). Opportunistic pathogens enriched in showerhead biofilms. Proc. Natl. Acad. Sci. USA.

[47] Francone A., Merino S., Retolaza A., Ramiro J., Alves S.A., de Castro J.V., Neves N.M., Arana A., Marimon J.M., Torres C.M.S. (2021). Impact of surface topography on bacterial attachment to micro- and nano-patterned polymer films. Surf. Interfaces.

[48] Zheng S., Bawazir M., Dhall A., Kim H.E., He L., Heo J., Hwang G. (2021). Implication of surface properties, bacterial motility, and hydrodynamic conditions on bacterial surface sensing and their initial adhesion. Front. Bioeng. Biotechnol..

[49] Brooke J.S., Annand J.W., Hammer A., Dembkowski K., Shulman S.T. (2009). Investigation of bacterial pathogens on 70 frequently used environmental surfaces in a large urban U.S. university. J. Environ. Health.

[50] Täubel M., Rintala H., Pitkäranta M., Paulin L., Laitinen S., Pekkanen J., Hyvärinen A., Nevalainen A. (2009). The occupant as a source of house dust bacteria. J. Allergy Clin. Immunol..

[51] Martens A., Schauer M., Motevalli M., König B. (2026). Patterns, associated factors, and anatomical concordance of nasal and throat *Staphylococcus aureus* carriage among community-dwelling adults in Germany. Microorganisms.

[52] Ben Maamar S., Hu J., Hartmann E.M. (2020). Implications of indoor microbial ecology and evolution on antibiotic resistance. J. Expo. Sci. Environ. Epidemiol..

[53] Sansom S.E., Gussin G.M., Schoeny M., Singh R.D., Adil H., Bell P., Benson E.C., Bittencourt C.E., Black S., Del Mar Villanueva Guzman M. (2024). Rapid environmental contamination with *Candida auris* and multidrug-resistant bacterial pathogens near colonized patients. Clin. Infect. Dis..

[54] Martens A., Motevalli M., Schauer M., Mair S., König B. (2026). Effectiveness of Hydrogen Peroxide Nebulization Combined with Manual Cleaning in Reducing Surface-Associated Microorganisms in an Indoor Fitness Environment. Microorganisms.

