# Supplementary material for: Serial Surveillance of Viable Bacterial Contamination in a German Hotel Across Pre-COVID and Pandemic Recovery Periods: Persistent Hotspots and Functional-Area Heterogeneity
Source: Microorganisms. 2026 Sep 29;14(10):2183. doi: 10.3390/microorganisms14102183 (PMC13707921; doi:10.3390/microorganisms14102183)

Supplementary Materials (*Table S1, Figure S1, Figure S2, and Figure S3*)

Table S1. Sensitivity, sampling comparability, and audit-year influence analyses of surveillance-period contrasts.

Outcome / environment	Scenario / condition	N	Events	Clusters / shared support	Pre-COVID	COVID-associated	Adjusted PR (95% CI)	Adjusted difference, pp (95% CI)	P value
A. Robustness of adjusted COVID-associated versus pre-COVID contrasts									
Positive culture	Primary four-area analysis	544	336	57	62.9%	60.7%	0.96 (0.83 to 1.12)	-2.2 (-11.5 to 7.1)	0.634
Positive culture	Exclude initial 2016 audit	442	268	46	60.6%	60.6%	1.00 (0.82 to 1.22)	-0.0 (-12.1 to 12.1)	1.000
Positive culture	Shared area-by-condition support	402	240	41	58.4%	60.3%	1.03 (0.84 to 1.27)	1.9 (-10.2 to 14.0)	0.755
Positive culture	Guest rooms sampled after cleaning	186	93	19	44.4%	51.3%	1.15 (0.82 to 1.64)	6.9 (-9.2 to 23.0)	0.417
Positive culture	Wellness/water only	95	43	6 *	51.0%	39.1%	0.77 (0.43 to 1.37)	-11.9 (-41.5 to 17.8)	0.371
Positive culture	Recurring guest-room surface types	200	86	30	43.1%	42.9%	0.99 (0.67 to 1.46)	-0.3 (-16.9 to 16.4)	0.974
Positive culture	Explicitly documented ESwab	501	299	57	61.0%	58.2%	0.96 (0.81 to 1.13)	-2.7 (-13.0 to 7.5)	0.601
Positive culture	Exclude QA review-flagged records	516	317	56	63.1%	60.0%	0.95 (0.81 to 1.11)	-3.1 (-12.7 to 6.4)	0.514
Positive culture	Alternative subarea clustering	544	336	66	62.9%	60.7%	0.96 (0.83 to 1.11)	-2.2 (-11.2 to 6.7)	0.623
High contamination	Primary four-area analysis	537	195	57	53.6%	19.5%	0.36 (0.25 to 0.54)	-34.1 (-46.6 to -21.5)	<0.001
High contamination	Exclude initial 2016 audit	440	132	46	46.4%	19.7%	0.42 (0.27 to 0.67)	-26.7 (-43.3 to -10.2)	<0.001
High contamination	Shared area-by-condition support	398	109	41	38.4%	20.6%	0.54 (0.31 to 0.92)	-17.8 (-34.8 to -0.7)	0.023
High contamination	Guest rooms sampled after cleaning	185	26	19	38.9%	8.1%	0.21 (0.06 to 0.67)	-30.8 (-52.3 to -9.3)	0.009
High contamination	Wellness/water only	95	29	6 *	42.9%	17.4%	0.41 (0.21 to 0.77)	-25.5 (-53.2 to 2.2)	0.006
High contamination	Recurring guest-room surface types	198	46	30	36.6%	9.3%	0.25 (0.07 to 0.86)	-27.4 (-45.3 to -9.4)	0.028

High contamination	Explicitly documented ESwab	494	177	57	53.2%	16.3%	0.31 (0.20 to 0.47)	-36.9 (-48.6 to -25.2)	<0.001
High contamination	Exclude QA review-flagged records	516	189	56	56.3%	19.3%	0.34 (0.24 to 0.50)	-37.0 (-48.7 to -25.3)	<0.001
High contamination	Alternative subarea clustering	537	195	66	53.6%	19.5%	0.36 (0.24 to 0.55)	-34.1 (-46.7 to -21.5)	<0.001
High contamination	Missing severity assigned non-high	544	195	57	52.1%	19.5%	0.37 (0.25 to 0.56)	-32.6 (-45.1 to -20.1)	<0.001
High contamination	Missing severity assigned high	544	202	57	54.3%	19.9%	0.37 (0.25 to 0.54)	-34.4 (-46.9 to -22.0)	<0.001
Priority microorganism	Primary four-area analysis	538	81	57	14.6%	15.5%	1.06 (0.67 to 1.67)	0.9 (-5.9 to 7.6)	0.804
Priority microorganism	Exclude initial 2016 audit	436	67	46	15.1%	15.5%	1.03 (0.56 to 1.89)	0.4 (-8.8 to 9.7)	0.926
Priority microorganism	Shared area-by-condition support	402	56	41	9.9%	16.3%	1.64 (0.92 to 2.93)	6.4 (0.1 to 12.7)	0.092
Priority microorganism	Guest rooms sampled after cleaning	186	24	19	8.3%	14.0%	1.68 (0.50 to 5.60)	5.7 (-5.4 to 16.7)	0.398
Priority microorganism	Wellness/water only	95	9	6 *	10.2%	8.7%	0.85 (0.29 to 2.53)	-1.5 (-12.6 to 9.6)	0.773
Priority microorganism	Recurring guest-room surface types	199	27	30	10.9%	16.3%	1.50 (0.60 to 3.75)	5.4 (-6.0 to 16.9)	0.386
Priority microorganism	Explicitly documented ESwab	495	72	57	14.8%	14.3%	0.97 (0.61 to 1.55)	-0.4 (-7.3 to 6.4)	0.900
Priority microorganism	Exclude QA review-flagged records	516	78	56	14.7%	15.5%	1.05 (0.65 to 1.70)	0.7 (-6.5 to 8.0)	0.842
Priority microorganism	Alternative subarea clustering	538	81	66	14.6%	15.5%	1.06 (0.68 to 1.66)	0.9 (-5.8 to 7.5)	0.800
Priority microorganism	Unidentified positive cultures assigned non-priority	544	81	57	14.3%	15.5%	1.09 (0.69 to 1.71)	1.2 (-5.4 to 7.8)	0.719
Priority microorganism	Unidentified positive cultures assigned priority	544	87	57	16.5%	15.5%	0.93 (0.59 to 1.49)	-1.1 (-8.6 to 6.5)	0.776
B. Sampling-condition support within functional environment and surveillance period									
Guest rooms	After cleaning	186	—	Yes	36	150	—	—	—
Guest rooms	Unoccupied room	97	—	No	97	0	—	—	—
Child-oriented areas	After cleaning	4	—	No	0	4	—	—	—

Child-oriented areas	Before operation	18	—	No	18	0	—	—	—
Child-oriented areas	Before operation/after cleaning	67	—	Yes	13	54	—	—	—
Child-oriented areas	During operation	23	—	No	23	0	—	—	—
Wellness/water	Before operation/after cleaning	95	—	Yes	49	46	—	—	—
Food service	During operation	26	—	Yes	14	12	—	—	—
Food service	During or after operation	28	—	Yes	18	10	—	—	—
C. Leave-one-audit-year-out influence analysis for culture positivity									
Positive culture	None (primary analysis)	544	336	57	62.9%	60.7%	0.96 (0.83 to 1.12)	-2.2 (-11.5 to 7.1)	0.634
Positive culture	Omit 2016	442	268	46	60.6%	60.6%	1.00 (0.82 to 1.22)	-0.0 (-12.1 to 12.1)	1.000
Positive culture	Omit 2018	473	305	46	70.1%	60.4%	0.86 (0.75 to 0.99)	-9.7 (-18.8 to -0.6)	0.033
Positive culture	Omit 2019	449	265	46	56.5%	60.7%	1.07 (0.90 to 1.28)	4.2 (-5.9 to 14.3)	0.424
Positive culture	Omit 2022	384	248	43	63.6%	66.7%	1.05 (0.86 to 1.28)	3.1 (-9.8 to 16.0)	0.637
Positive culture	Omit 2023	428	258	47	62.4%	56.5%	0.91 (0.78 to 1.06)	-5.9 (-15.1 to 3.3)	0.204

CI, confidence interval; CFU, colony-forming units; HC1, heteroskedasticity-consistent covariance estimator; pp, percentage points; PR, prevalence ratio; QA, quality assurance. **Values:** Pre-COVID and COVID-associated columns report adjusted prevalence percentages in sections A and C and sample counts in section B. Adjusted differences are COVID-associated minus pre-COVID prevalence. Section B contains counts rather than model estimates. **Models:** Sections A and C use modified Poisson regression with a log link and HC1 cluster-robust covariance. Default clusters are audit-year-by-source-table combinations; the alternative clustering scenario uses audit year by environment by subarea. Models spanning multiple environments adjust for functional environment; single-environment models include the surveillance-period term only. Confidence intervals are 95%, and two-sided Wald P values are exploratory without multiplicity adjustment across sensitivity scenarios. **Outcome handling:** High contamination denotes >500 CFU or confluent growth. Primary high-contamination analyses exclude seven samples with unavailable severity; the two bounding scenarios classify all seven as non-high or high. Primary priority-microorganism analyses exclude six unidentified positive cultures; the corresponding bounds classify all six as non-priority or priority. **Support and stability:** *Fewer than 10 independent clusters; corresponding confidence intervals and P values require caution. Green-shaded section-B rows identify environment-by-condition combinations observed in both surveillance periods and retained in the shared-support analysis (402 of 544 samples). Blue shading identifies primary analyses; purple shading identifies missing data bounds. **Interpretation:** The leave-one-year-out analysis evaluates dependence on individual audit campaigns and does not estimate a continuous temporal trend.

Figure S1. Complete ordinal contamination-severity distributions across audit years, surveillance periods, and functional environments.

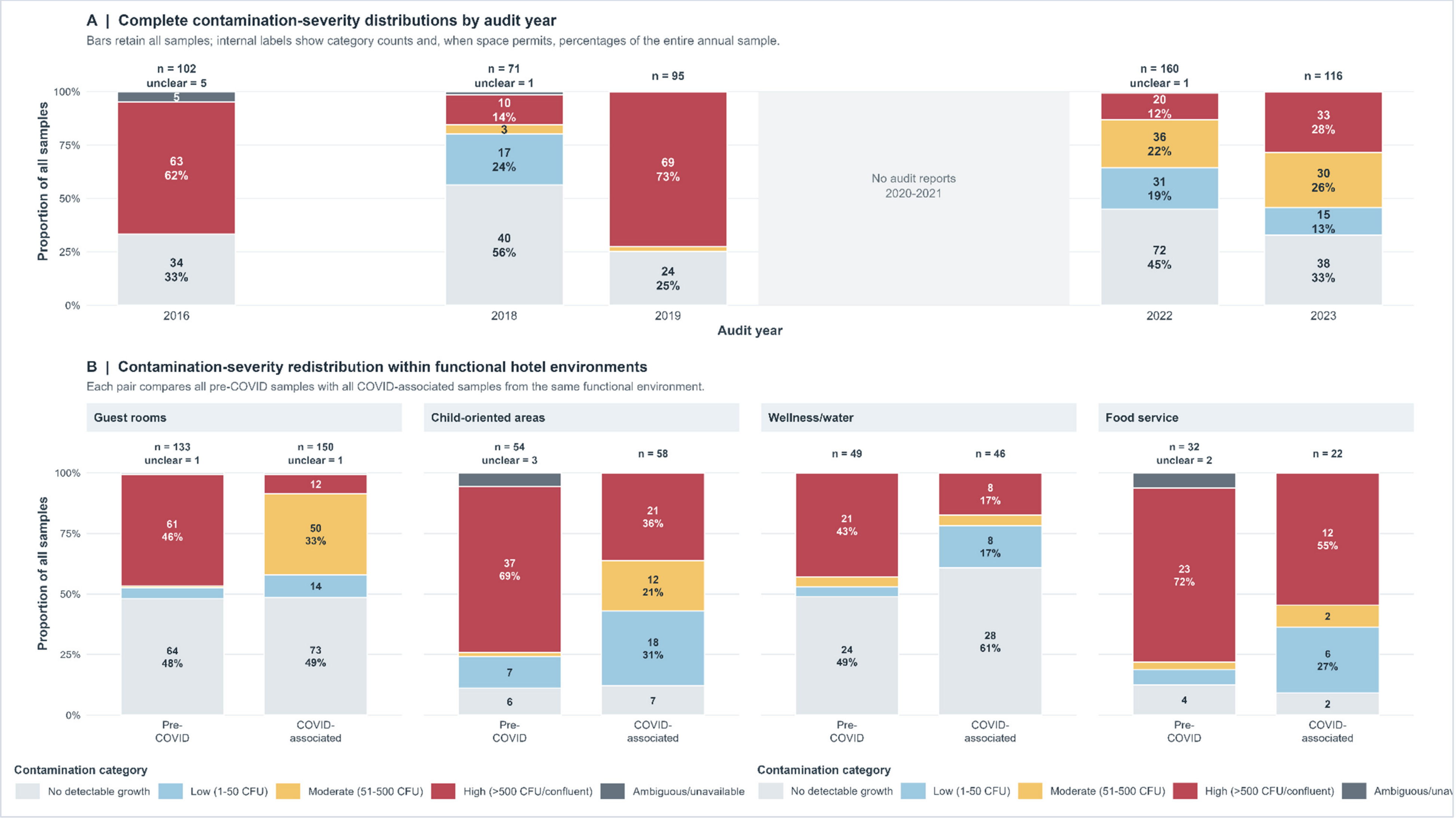

Figure S2. Sensitivity analyses, sampling comparability, and audit-year influence on surveillance-period associations.

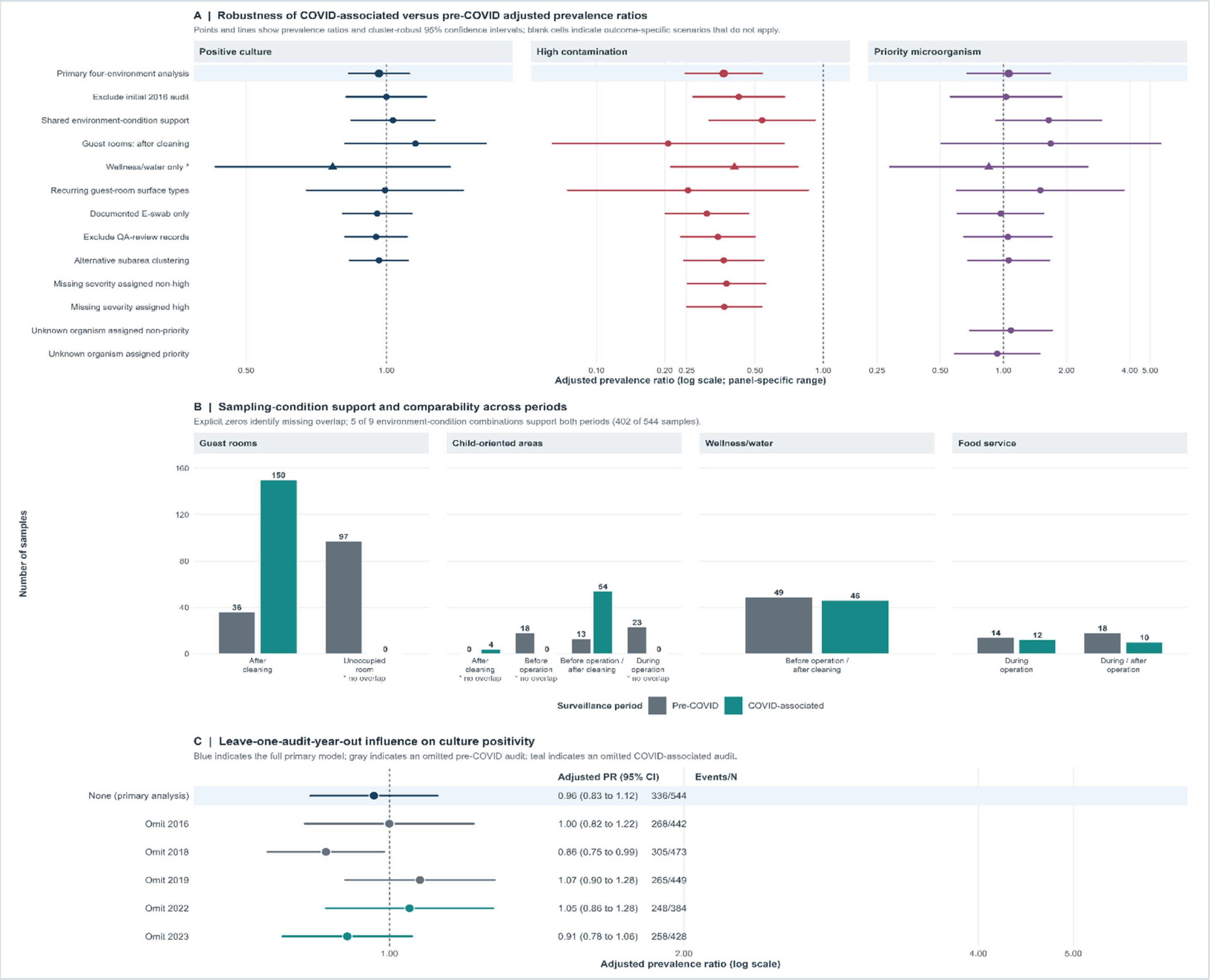

Figure S3. Environmental hygiene outcomes in child-oriented areas and across child-use surface classes.

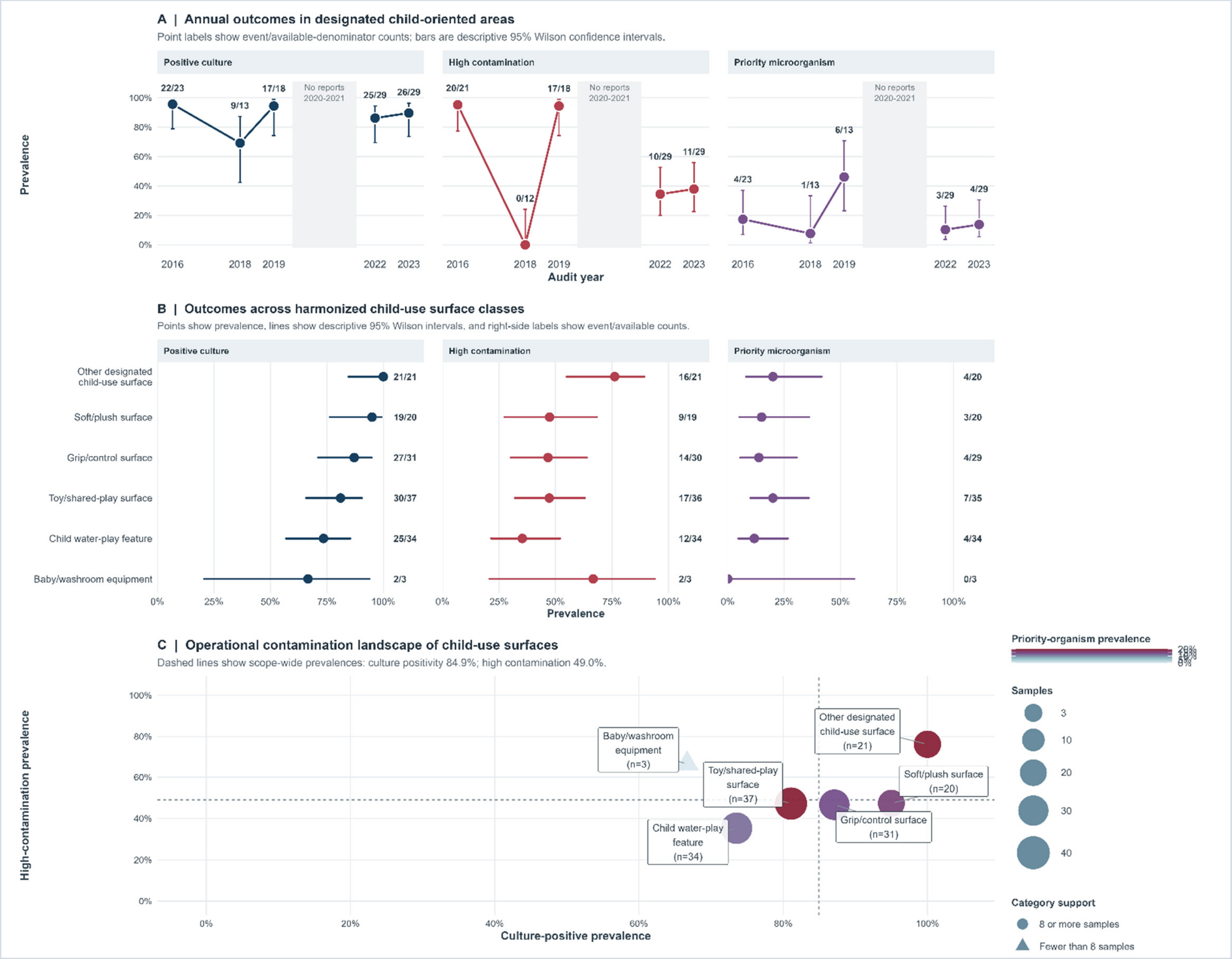

Supplement: Supplementary file 1 [file microorganisms-14-02183-s001.zip › microorganisms-4575933-supplementary.pdf]
